# Traditional Chinese medicine formula Bi-Qi capsule alleviates rheumatoid arthritis-induced inflammation, synovial hyperplasia, and cartilage destruction in rats

**DOI:** 10.1186/s13075-018-1547-6

**Published:** 2018-03-14

**Authors:** Kai Wang, Dongmei Zhang, Yan Liu, Xuan Wang, Jiantong Zhao, Tingting Sun, Tingting Jin, Baoli Li, Janak L. Pathak

**Affiliations:** 1 Department of International Medicine, Geriatric Disease Research Institute, Tianjin Hospital, Tianjin, 300211 China; 2 Department of Traditional Chinese Medicine, Tianjin Medical University General Hospital, Tianjin, 300070 China; 3 School of Pharmaceutical Science and Technology, Health Sciences Platform, Tianjin University, Tianjin, 300072 China

**Keywords:** Rheumatoid arthritis, Bi-Qi-capsule, Pathogenesis, Inflammation, Synovial hyperplasia, Cartilage destruction, Methotrexate

## Abstract

**Background:**

Traditional Chinese medicine (TCM) formula Bi-Qi capsule (Bi-Qi) is a commonly prescribed drug to treat rheumatoid arthritis (RA). However, the mechanism of Bi-Qi-mediated amelioration of RA pathogenesis is still a mystery. Collagen induced arthritis (CIA) in rats is an established model that shares many similarities with RA in humans. In this study we investigated the effect of Bi-Qi on the pathogenesis of CIA in rats.

**Methods:**

CIA was developed in Sprague-Dawley (S.D) rats (*n* = 60, female) and used as a model resembling RA in humans. Rats were treated with a high or moderate dose of Bi-Qi, or methotrexate (MTX). Effects of the treatment on local joint and systemic inflammation, synovial hyperplasia, cartilage destruction, and other main features in the pathogenesis of CIA were analyzed.

**Results:**

Inflamed and swollen ankles and joints were observed in arthritic rats, while Bi-Qi or MTX treatment alleviated these symptoms. Only the Bi-Qi moderate dose decreased RA-induced serum levels of tumor necrosis factor-alpha (TNF-α). Both Bi-Qi and MTX reduced the interleukin (IL)-18 serum level. Protein levels of cartilage oligomeric matrix protein and osteopontin in serum, synovium, and cartilage were elevated in arthritic rats, while Bi-Qi alleviated these effects. Synovial hyperplasia, inflammatory cell infiltration in synovium and a high degree of cartilage degradation was observed in RA, and Bi-Qi or MTX alleviated this effect. Bi-Qi at the moderate dose was the most effective in mitigating CIA-related clinical complications.

**Conclusions:**

Our findings showed that Bi-Qi alleviates CIA-induced inflammation, synovial hyperplasia, cartilage destruction, and the other main features in the pathogenesis of CIA. This provides fundamental evidence for the anti-arthritic properties of Bi-Qi and corroborates the use of Bi-Qi TCM formula for the treatment of RA.

**Electronic supplementary material:**

The online version of this article (10.1186/s13075-018-1547-6) contains supplementary material, which is available to authorized users.

## Background

The Bi-Qi capsule is a TCM formula approved by the Chinese Food and Drug Administration to treat rheumatoid arthritis (RA), cervical spondylosis, scapulohumeral periarthritis, and knee osteoarthritis [1–5]. *Radix Salviae miltiorrhizae* (Dan Shen), *Semen strychni* (Ma Qian Zi), *Radix glycyrrhizae* (Gan Cao), Radix *Codonopsis pilosula* (Dang Shen), *Astragalus membranaceus* (Fisch), Bunge (Huang qi), Notoginseng (San Qi), *Rhizoma Ligustici chuanxiong* (Chuan Xiong), *Radix Achyranthis bidentata* (Huai Niu Xi), and *Rhizoma Atractylodis macrocephalae* (Bai Zhu) are Chinese herbal ingredients of the Bi-Qi capsule. Tanshinone IIA sulfonic sodium, salvianolic acid B, glycyrrhizin, brucine, strychnine, cryptotanshinone, and liquiritin are major compounds present in Bi-Qi extract [6, 7]. Tanshinone IIA sulfonic sodium is a water-soluble extract from *Radix et Rhizoma S. miltiorrhizae* with a strong anti-osteoporotic property [8]. Bi-Qi has been shown to have anti-inflammatory and analgesic properties [3]. However, the mechanism of Bi-Qi-mediated amelioration of RA pathogenesis is still a mystery.

RA is a chronic systemic autoimmune disease which primarily involves synovial joint pain, immobility, and stiffness. RA exhibits extreme variation, ranging from mild, self-limiting disease to rapidly progressive arthritis with extra-articular manifestations [9, 10]. Focal marginal articular erosions, subchondral bone loss, periarticular osteopenia, systemic inflammation, and osteoporosis are the main pathologic stages of skeletal remodeling that characterize RA. RA-related systemic complications affect major vital organs including heart, lung, liver, brain, and bone [11]. Unknown etiology and doubtful prognosis are the main challenges to treating RA. RA initially affects freely movable joints such as joints in the hand, shoulder, knee, and hip. Macrophages, plasma cells, and lymphocytes infiltrate the synovium causing synovial hyperplasia. Increased inflammatory immune cells and fibroblast-like synoviocyte infiltration form pannus and small blood vessels that lead to synovium and cartilage destruction [12, 13]. These cells produce various cytokines and chemokines; among these, TNF-α and IL-18 have been reported to play important role in the pathogenesis of RA [14–16]. Inflamed synovium in RA produces larger amounts of osteopontin (OPN) [17, 18]; it is a pro-inflammatory protein with a critical role in leukocyte migration and production of IL-17 from T cells, thereby play a key role in the pathogenesis of RA [17]. The cartilage oligomeric matrix protein (COMP) is an extracellular matrix protein mainly localized to tendon, cartilage, and pericartilage tissues [19]. Serum and synovial fluid COMP levels are reported to be promising RA diagnostic and prognostic markers [19, 20]. Therefore, TNF-α, IL-18, OPN, and COMP are important markers in investigating the pathogenesis of RA.

Nonsteroidal anti-inflammatory drugs, corticosteroids, disease-modifying anti-rheumatic drugs (DMARDs), and biological agents such as anti-TNF-α and anti-IL-6-receptor antibodies are drugs commonly prescribed to treat RA. However, these drugs have adverse side effects such as bone loss, liver failure, respiratory failure, dermatological damage, and risk of infection [21, 22]. Methotrexate (MTX) is the most commonly used DMARD. Due to its perceived efficacy, acceptable safety profile, low cost, and decades of clinical experience, MTX remains the initial preferred drug and is considered to be the gold standard for treatment of RA [23]. Moreover, pathophysiological and genetic differences among patients also limit the therapeutic effects of these drugs in RA treatment. Compared with the aforementioned conventional Western drugs, TCM provides a more flexible approach to treat RA because various combinations, dosages, and compatibility of herbs are modified according to the pathophysiological condition of the individual patient. TCM formulas have been used for 3000 years, with efficacy to treat disease and neutralize the toxic effects of herbal components in the mixture. Many TCM formulas are in use to treat RA. Bi-Qi is the most commonly used effective TCM formula to treat RA with the least adverse effects. However, the mechanism behind Bi-Qi-mediated mitigation of RA pathogenesis has not yet been investigated.

In this study, we aimed to analyze whether Bi-Qi modulates RA-induced inflammation, synovial hyperplasia, and cartilage destruction. We used a CIA rat model that resembles RA in humans. We treated the CIA with Bi-Qi using two different doses or with MTX. MTX was used as a positive control for RA treatment. Interestingly, Bi-Qi at the moderate dose alleviated the pathogenesis of CIA, i.e., local and systemic inflammation, infiltration of immune cells in synovium, synovium hyperplasia, and cartilage destruction. Bi-Qi moderate dose reduced paw swelling and the arthritic score.

## Methods

### CIA model

The CIA rat model shares many similarities with RA in humans and is widely used for RA-related in vivo studies. Rats are much larger than mice and provide easy visual analysis of the effect of CIA and treatment against CIA in the ankle and joints. Sprague-Dawley (S.D) rats are an established strain for CIA. Female S.D rats (*n* = 60, 4 weeks old, 150 ± 10 g in weight) were bought from the Animal Center of Tianjin Military Sciences Academy. Ten rats were randomly selected as control (control group), and CIA was developed in the other 50 rats with slight modification to previously described techniques [24, 25]. Briefly, 10 mg of type II freeze-dried collagen was mixed with 0.05 ml acetic acid and was constantly swung for 12 h at 4 °C to obtain a 2 mg/ml collagen solution. The collagen solution was mixed with incomplete Freund adjuvant (1:1 volume ratio) to obtain a collagen emulsion. The collagen emulsion (0.2 ml) was injected into the rat-tail root. Rats in the control group were injected with an equal volume of saline. At day 7, another 0.1 ml of collagen emulsion was injected into the rat-tail root avoiding the original pinholes. Rats in the control group were again injected with equal volume saline. Animals were housed and acclimatized under standard rat chow diet and tap water under climate-controlled conditions (25 °C; 55% humidity, 12-h light/12-h darkness). All procedures followed were in accordance with the ethical standards of Tianjin Medical University Animal Care and Use Committee guidelines. The study protocol was approved by Animal Experimental Ethical Panel of Tianjin Medical University.

### Bi-Qi-extract or MTX treatment

After 2 weeks of collagen emulsion treatment, the arthritis score was measured as described previously [26]. Briefly, a score of 0 represents no red skin and swollen joint symptoms, a score of 1 represents red and slightly swollen ankle or foot, a score of 2 represents red and slightly swollen ankle and mid foot, a score of 3 represents red and moderately swollen ankle and metatarsal joint, and a score of 4 represents red and severely swollen whole ankle, foot and foot plantar. In addition, tail erythema and swelling of the forelimb each score 0.5, which makes a total arthritic score of 5. The severity of arthritis was assessed by visual observation by three independent observers. The observers were unaware about the treatment groups. Paw swelling rate was analyzed every 2 days till day 42 using a water displacement method and a plethysmometer (YLS-7B, Ji’nan Yi Yan science and Technology Development Co., Ltd., Jinan, China) as described previously [27]. Paw swelling rate was measured in both hind limb and average per limb was calculated. Among 50 collagen-treated rats, 43 rats developed severe arthritis (arthritic score >4). Forty rats with an arthritis score >4 were randomly selected and divided into four different groups (ten rats in each group): arthritic group, arthritic rats treated with a high dose of Bi-Qi extract (HDBQ group), arthritic rats treated with a moderate dose of Bi-Qi extract (MDBQ group), and arthritic rats treated with MTX (MTX group). MTX is a drug commonly used to treat rheumatoid arthritis [28] and was therefore used as a positive control in this study. Normal saline in the control group and 0.9 g/kg body weight/day Bi-Qi extract in the HDBQ group or Bi-Qi extract 0.6 g/kg body weight/day in the MDBQ group was gavaged for 4 weeks. In the MTX group, MTX 1.67 mg/kg body weight/week was injected subcutaneously into the abdomen. The dose of MTX was chosen based on the literature [29–31]. Since the dose of MTX mentioned in the literature and used in this study was ~ 10-fold higher than clinical dose, we choose 10-fold and 15-fold higher doses of Bi-Qi. In clinical practice diarrhoea is a commonly observed side effect of Bi-Qi. In this study we also noted occasional diarrhoea in Bi-Qi treated rats. Walking gait, mental alertness, hair color, diet, and stool were observed every 2 days as a physiological and behavioral observation. Body weight was measured every week. Two untreated rats in the arthritic group died at week 5 possibly due to disease severity. Therefore, eight rats were randomly selected from each group to analyze the effect of the treatment.

### Serum and tissue collection

Once CIA was established, 2 ml blood was collected from inner canthus. After 4 weeks of treatment with Bi-Qi or MTX, the rats were anesthetized using isofluorane. Blood was collected (7–8 ml) from the abdominal aorta and serum was separated within 1 h of blood collection, by centrifugation for 15 min at 1.500 *g*. The serum was stored without preservative at − 80 °C and then thawed just prior to testing. The rats were killed after blood collection, and skin, fascia, and muscles around the ankles were removed. The left ankles (with the bone) were fixed in 4% paraformaldehyde (PFA) for 72 h. The right ankles were quickly frozen in liquid nitrogen.

### Histology and immunohistochemistry assessment

The left ankles were decalcified according to the Plank-Rychlo protocol. Synovium was selected at the ankle joint and cartilage was selected on the surface of the trochleae at ankle joint. For proper tissue selection, synovium and cartilage were confirmed by microscopic morphology study. The PFA fixed tissues were dehydrated in an ascending series of ethanol and embedded in paraffin. Tissue sections of 6-μm thickness were prepared using a rotary microtome (Huiwo Science and Technology Co. Ltd., Shenzhen, China). To analyze the pathological morphology of synovium and cartilage, histological sections were stained with hematoxylin and eosin and pictures were taken at magnification × 400 using a light microscope (Olympus, Japan). For semi-quantitative analysis, five random microscopic fields/images from five random images/rat were analyzed by Image Pro Plus 6.0.

Pathological features of the synovial membranes were analyzed by semi-quantification of inflammatory cell infiltration, synovial cell proliferation, and proliferation of fibrous tissue in the synovium. Neutrophil infiltration was classified as 0–2, i.e., 0, no cells; 0.5, 1–5 cells/high power magnification field (HPF); 1, 6–10 cells/HPF; 1.5, 11–15 cells/HPF; or 2, micro abscess. Lymphocyte infiltration was classified as 0–2 based on number of cells present/HPF. Plasma cells infiltration was classified as 0–2, i.e., 0.5, 1–5 cells/HPF; 1, 6–10 cells/HPF; 1.5, 11–15/HPF; or 2, > 15 cells/HPF. Proliferation of fibrous tissue was classified as 0–2 based on the area of fibrous tissue/HPF. Synovial cell proliferation was classified as 0–2, i.e., 0, no synovial cell infiltration in synovium, 0.5: swollen synovium, 1: swollen synovium with few cells infiltration; 1.5, synovial infiltration in two layers of synovium; 2, synovial infiltration in > 3 layers.

The depth of the damage to the cartilage layer was classified as 0–3, i.e., 0, smooth and intact and smooth cartilage layer; 1, superficial damage of cartilage layer; 2, damage to half of the depth of the cartilage layer; 3, damage of full depth of cartilage layer. The range of damage at the cartilage surface was classified as 0–3, i.e., 0, no damage on cartilage surface; 1, focal damage; 2, > 1/3 to < 2/3 of cartilage surface damage; 3, > 2/3 of cartilage surface damage. Similarly proliferation of fibrous tissue was classified as 0–3 based on the degree of proliferation. Furthermore, inflammatory cell infiltration in the cartilage was classified as 0–3 based on the number of infiltrated cells.

### COMP and OPN expression

To analyze COMP and OPN expression in synovium tissue, tissue sections were rehydrated and endogenous peroxidase was quenched with 3% H_2_O_2_ in PBS with 40% methanol. Antigen retrieval was performed by 20-min incubation with 10 mM citrate buffer at room temperature. After blocking of non-specific binding sites with 10% normal goat serum for 1 h, the sections were incubated overnight at 4 °C with mouse-anti-COMP antibody (Boiss antibodies company, Beijing, China) in PBS or mouse anti-OPN antibody (Boster Bioengineering Co.Ltd., Wuhan, China) in PBS. The sections were incubated for 1 h with 1/100 biotinylated secondary rabbit anti-mouse antibody (ZhongshanJinqiao Bioengineering Co. Ltd., Beijing, China) and for 1 h with horseradish peroxidase-labeled streptavidin (Zhongshan Jinqiao Bioengineering Co. Ltd., Beijing, China). For color development, the sections were incubated with DAB reagent (Zhongshan Jinqiao Bioengineering Co. Ltd., Beijing, China) and counterstained with hematoxylin and pictures were taken at × 400 magnification using a light microscope. For semi-quantitative analysis, five random microscopic fields/images from five random images/rat were analyzed by Image Pro Plus 6.0.

### Enzyme-linked immunosorbent assay (ELISA) for OPN, COMP, TNF-α and IL-18

Quantification of OPN, COMP, TNF-α and IL-18 in serum samples was performed using ELISA kits (R&D systems) following the manufacturer’s protocol. The minimum detection limit was 5 pg/ml for TNF-α, 5.7 pg/ml for OPN, and 10 pg/ml for IL-18 and COMP.

### RNA isolation and quantitative real-time PCR analysis

Cartilage tissue (around 10 mg) frozen at − 80 °C was crushed in a mortar in the presence of liquid nitrogen. Total RNA was isolated using Trizol (Sango Biotech Co. Ltd., Shanghai, China). The concentrations of isolated RNAs were determined using a TECAN Infinite® F200 Pro microtiterplate reader (Thermo Fisher Scientific). cDNA synthesis was performed with 6 μl of total RNA in 20 μl of reaction volume using RevertAid First Strand cDNA Synthesis Kit (Sango Biotech Co. Ltd., Shanghai, China) according to the manufacturer’s instructions.

Quantitative expression of COMP and OPN was measured using two-step reverse transcription PCR. Real time PCR reactions were performed using 1 μl cDNA and Hotstart Fluo-PCR mix (Sango Biotech Co. Ltd., Shanghai, China) according to the manufacturer’s instructions. The determined cycle threshold (CT) values of the target genes were normalized to the relative expression of the housekeeping gene *Gapdh*. The primer sequence for COMP was forward: 5’-GTGGTGCTCAATCAGGGAAT-3′, reverse: 5′-G AAGCCAGCGTAGTCATCATC-3′; for OPN it was forward: 5’-AAGCGTGGAAACACACAGC-3′, reverse: 5’-TTTGGAACTCGCCTGACTG-3′; and for GAPDH it was forward: 5’-CAACTCCTCAAGATTGTCAGCAA-3′, reverse: 5’-GGCATGGACTGTGGTCATG A-3′.

### Statistical analysis

Data are presented as mean ± SD. Effects of different treatments were tested using analysis of variance (ANOVA) followed by Sidak’s multiple comparison post hoc test. Differences were considered significant if *P* was < 0.05. Statistical analysis was performed using GraphPad Prism 5.01 (GraphPad Software, Inc., La Jolla, CA, USA).

## Results

### Collagen treatment successfully induced RA, and Bi-Qi treatment alleviated CIA-related symptoms

**Fig. 1:**
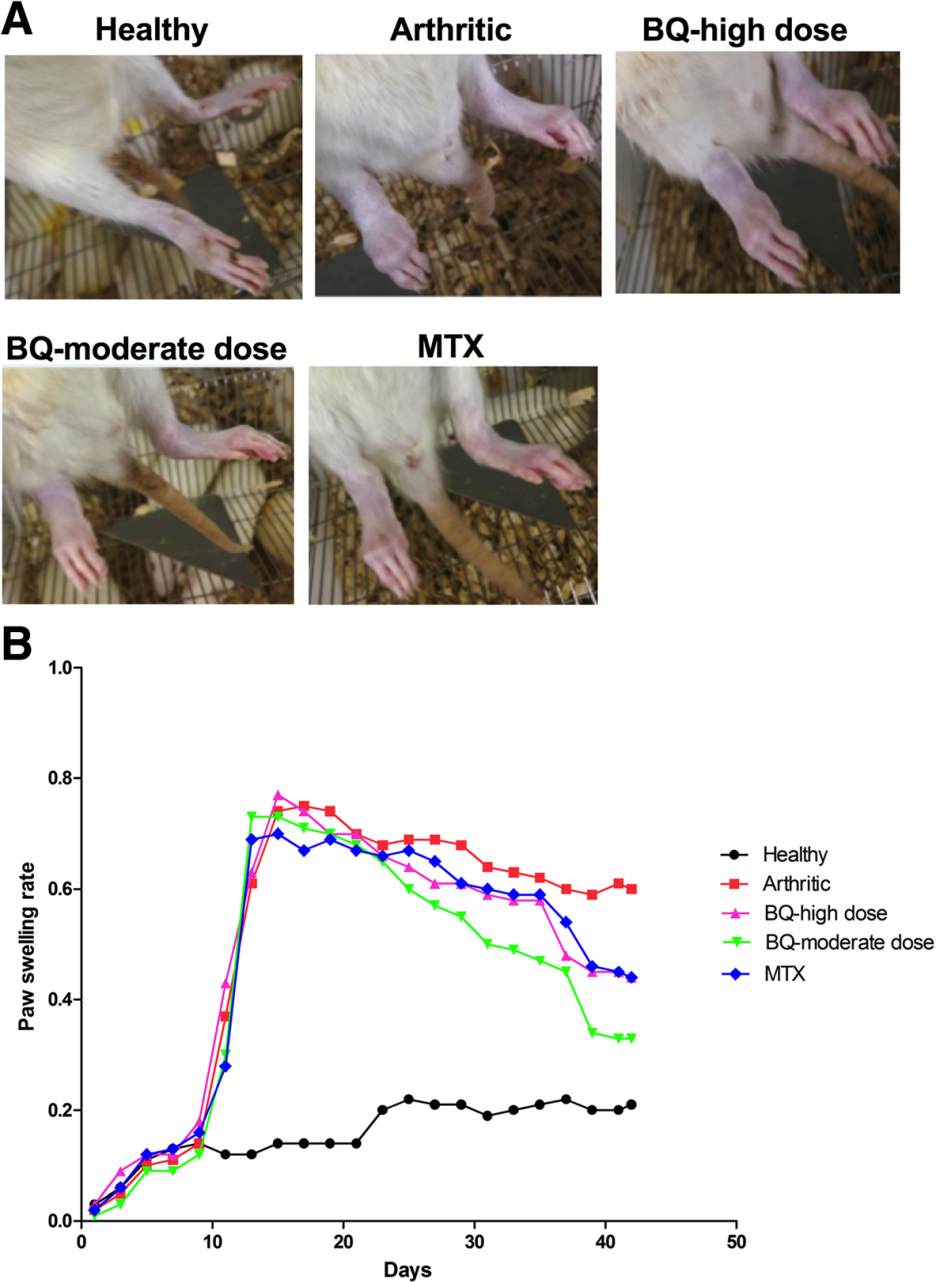
Bi-Qi capsule or methotrexate (MTX) alleviated paw swelling in collagen induced arthritis (CIA). **a** Representative paws from rats under different treatment conditions at week 6. **b** Quantitative analysis of paw swelling rate. Data shown are the average value/hind limb from eight rats in each group

Collagen treatment for 2 weeks induced RA in the rats. Rat paws were inflamed and small joints were swollen in rats in the arthritic group (Fig. 1a). The moderate-dose Bi-Qi capsule alleviated arthritis-induced paw inflammation and joint swelling more effectively compared to the high dose Bi-Qi capsule (Fig. 1a, b). The paw swelling rate at day 40, 41, and 42 was dramatically reduced in the Bi-Qi moderate dose group compared to the other groups tested (Fig. 1b). Physiological and behavioral observation identified the negative effect of CIA on walking gait, mental alertness, hair color, and diet. Bi-Qi or MTX treatment slightly alleviated this effect (Additional file 1: Table S1). Slightly loose stool was observed in Bi-Qi or MTX treated groups (Additional file 1: Table S1). Rats in the healthy group had higher body weight at different time points compared to all other rats in the treated and untreated arthritic groups (Additional file 1: Table S2). There was no difference in body weight among the treated and untreated rats in the arthritic groups (Additional file 1: Table S2). Physical and behavioral observation showed no adverse effect of Bi-Qi treatment, except occasional diarrhoea.

The arthritic score was > 4 in the arthritic group from week 2 to 6. Three weeks of treatment with Bi-Qi or MTX did not significantly reduce the arthritic score. Interestingly, 4 weeks of high-dose Bi-Qi, moderate-dose Bi-Qi, and MTX treatment reduced the arthritic score by 13%, 22%, and 15% respectively (Table 1).

**Table 1:** Arthritic score

Group (*n* = 8)	Week 2	Week 3	Week 4	Week 5	Week 6
Healthy	0	0	0	0	0
Arthritic	4.38 ± 0.35	4.50 ± 0.53	4.44 ± 0.32	4.38 ± 0.44	4.25 ± 0.27
BQ high dose	4.44 ± 0.42	4.56 ± 0.42	4.25 ± 0.46	4.13 ± 0.44	3.69 ± 0.37^***^
BQ moderate dose	4.50 ± 0.46	4.38 ± 0.35	4.19 ± 0.53	3.94 ± 0.32	3.31 ± 0.26^****, &^
MTX	4.44 ± 0.42	4.44 ± 0.32	4.31 ± 0.26	4.06 ± 0.56	3.63 ± 0.35^***^

Data are presented as mean ± SD

*BQ* Bi-Qi capsule, *MTX* methotrexate

Significant effect of treatment compared to arthritic group, ^***^P < 0.001, ^****^P < 0.0001; compared to BQ high-dose and MTX groups, ^**&**^*P < 0.05*

### Bi-Qi alleviated CIA-induced OPN and COMP expression in synovium, cartilage, and serum

**Fig. 2:**
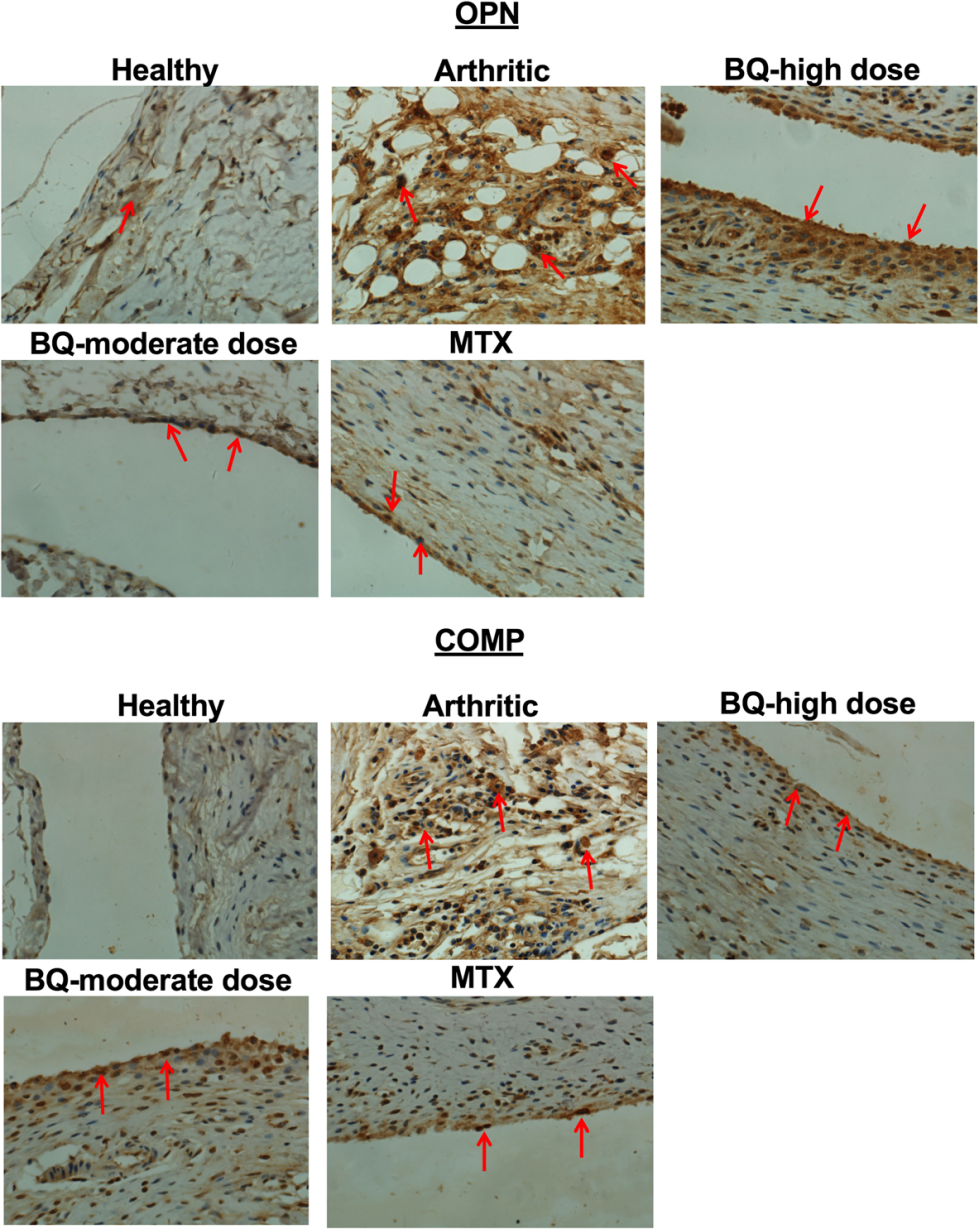
Collagen-induced arthritis (CIA) enhanced osteopontin (OPN) and cartilage oligomeric matrix protein (COMP) expression in paw joint cartilage, while the Bi-Qi capsule or methotrexate (MTX) treatment reduced this effect. Representative immunohistochemistry images of paw joint synovium with OPN and COMP immunostaining

**Fig. 3:**
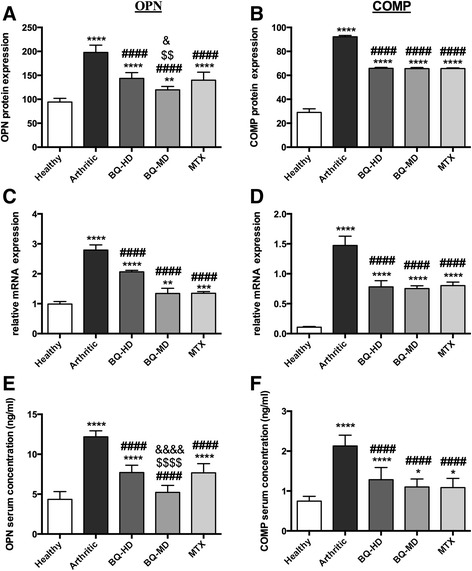
Bi-Qi capsule (BQ) or methotrexate (MTX) reduced collagen-induced arthritis (CIA)-induced osteopontin (OPN) and cartilage oligomeric matrix protein (COMP) upregulation in serum, and mRNA and protein levels in paw joint tissue. **a, b** OPN and COMP levels in serum. **c, d** OPN and COMP mRNA expression in paw joint cartilage. **e, f** OPN and COMP protein quantification from immunohistochemistry images of paw joint synovium. Data are presented as mean ± SD from eight rats in each group. Significant effect of treatment compared to control: *P < 0.05, **P < 0.01, ***P < 0.001, ****P < 0.0001. Significant effect of treatment compared to arthritic group: ^***####***^*P < 0.0001*. Significant effect of treatment compared to high-dose BQ (BQ-HD): ^*$$*^*P < 0.01,* ^*$$$$*^*P < 0.0001*. Significant effect of treatment compared to MTX-group: ^***&***^*P < 0.05,* ^*&&&&*^*P < 0.0001. BQ-MD, moderate-dose BQ*

Immunohistochemistry images of the synovium clearly indicated greater expression of OPN and COMP protein in the arthritic group compared to the healthy group (Fig. 2). High-dose or moderate-dose Bi-Qi treatment, or MTX treatment decreased the arthritis-induced OPN and COMP level in synovium (Fig. 2). Results from quantitative analysis showed that OPN expression in the arthritic synovium was increased by 110% compared to synovium in the healthy group (Fig. 3a). OPN expression was reduced by 27%, 40%, and 29%, respectively in the high-dose Bi-Qi, moderate-dose Bi-Qi, and the MTX groups compared to the arthritic group (Fig. 3a). Moderate dose Bi-Qi reduced OPN expression by 17% compared to high-dose Bi-Qi (Fig. 3a). Similarly, COMP expression was 217% higher in the arthritic synovium compared to that in healthy synovium (Fig. 3b). COMP expression was reduced by 28%, 29%, and 28.5%, respectively, in the high-dose Bi-Qi, moderate-dose Bi-Qi, and MTX groups compared to the arthritic group (Fig. 3b).

The mRNA expression levels of OPN and COMP were measured in cartilage. OPN expression in arthritic cartilage was 180% higher compared to healthy cartilage. Bi-Qi treatment at high dose, moderate dose, and MTX treatment reduced cartilage OPN expression by 28%, 53%, and 51% respectively, compared to the arthritic group (Fig. 3c). COMP expression in arthritic cartilage was 1400% higher compared to in healthy cartilage. Bi-Qi treatment at high dose, Bi-QI at moderate dose, and MTX treatment reduced cartilage COMP expression by 47%, 49%, and 45%, respectively, compared to the arthritic group (Fig. 3d).

Serum levels of COMP and OPN were measured by ELISA. COMP and OPN serum level in the arthritic group was 184% higher compared to that in the healthy group (Fig. 3e). Bi-Qi high dose, Bi-Qi moderate dose, and MTX treatment reduced serum OPN level by 37%, 57%, and 37%, respectively, compared to the arthritic group. Serum OPN level in the moderate-dose Bi-Qi group was 32% less compared to the high-dose Bi-Qi group (Fig. 3e). Bi-Qi treatment at high dose, Bi-Qi at moderate dose, and MTX treatment reduced serum COMP by 40%, 48%, and 40%, respectively, compared to the arthritic group (Fig. 3f).

### Bi-Qi moderate dose decreased arthritis-induced TNF-α and IL-18 serum concentration

Serum concentration of TNF-α was analyzed after 2 weeks of collagen treatment and after 4 weeks of Bi-Qi or MTX treatment. CIA elevated serum TNF-α concentration by 90% and 67% at week 2 and 6, respectively (Table 2). TNF-α serum level was 20% lower in the moderate-dose Bi-Qi group compared to the arthritic group. Bi-Qi treatment at high dose or MTX treatment for 4 weeks did not reduce the CIA-induced TNF-α upregulation (Table 2). Serum concentration of IL-18 was measured at after 4 weeks of Bi-Qi or MTX treatment. CIA increased IL-18 serum concentration by 25% at week 6 compared to the healthy group (Table 2). Bi-Qi high-dose treatment for 4 weeks did not affect serum IL-18 level. Bi-Qi treatment at moderate dose or MTX treatment for 4 weeks reduced serum IL-18 level by 17% compared to the arthritic group (Table 2).

**Table 2:** Serum levels of TNF-α and IL-18

Group (*n* = 8)	TNF-α (pg/ml)		IL-18 (pg/ml) week 6
	week 2	week 6	
Healthy	22.71 ± 1.79	23.87 ± 5.11	30.28 ± 1.76
Arthritic	43.15 ± 1.57^****^	39.80 ± 4.38^****^	38.03 ± 3.35^**^
BQ-high dose	43.35 ± 1.84^****^	34.04 ± 5.22^**^	33.69 ± 4.36
BQ-moderate dose	42.68 ± 2.30^****^	31.87 ± 2.88^*, ∆^	32.37 ± 4.20^∆^
MTX	43.28 ± 2.29^****^	33.84 ± 4.39^**^	32.32 ± 3.61^∆^

Data are presented as mean ± SD

*BQ* Bi-Qi capsule, *MTX* methotrexate

Significant effect of treatment compared to control, **P < 0.01, ****P < 0.0001; compared to arthritic group, ^∆^*P < 0.05*

### Bi-Qi inhibited arthritis-induced infiltration of inflammatory cells in synovium and synovial hyperplasia

**Fig. 4:**
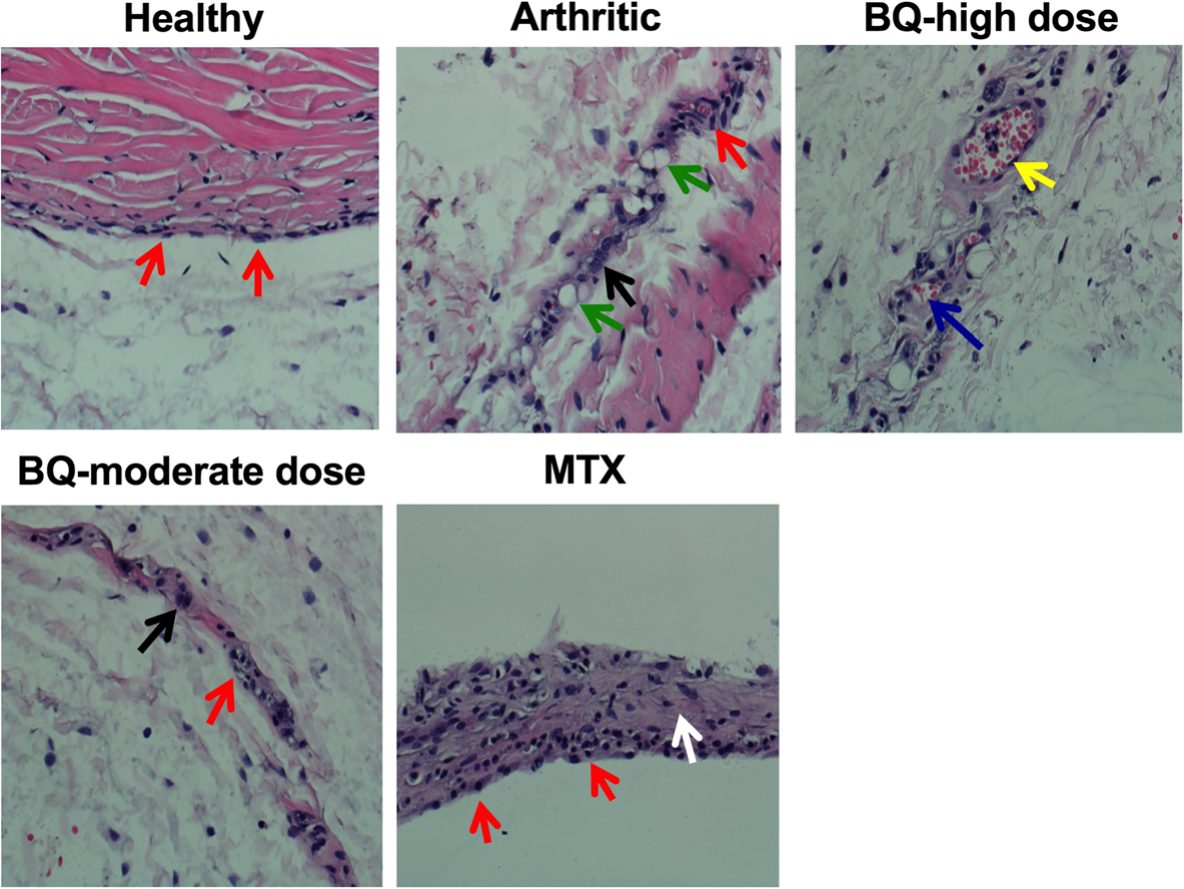
Bi-Qi capsule (BQ) or methotrexate (MTX) alleviated collagen-induced arthritis (CIA)-induced synovial membrane hyperplasia. Representative histological images of synovial membrane from the paw joint with H&E staining. Red arrow, synoviocyte; black arrow, plasmocyte; green arrow, lymphocyte; yellow arrow, pannus; blue arrow, vascular endothelial cells; white arrow, fibrous tissue growth

Healthy group synovium histology assessment showed an intact layer of synovial cells without inflammatory cell infiltration and synovial hyperplasia (Fig. 4). Disrupted synovial cell layering with inflammatory cell infiltration and synovial hyperplasia were observed in the arthritic group (Fig. 4). Bi-Qi treatment at moderate dose more efficiently alleviated arthritis-induced inflammatory cells infiltration in synovium and synovial hyperplasia compared to Bi-Qi treatment at high dose (Fig. 4).

Quantitative analysis showed that CIA caused a high extent of neutrophil, lymphocyte and plasma cell infiltration in synovium. Bi-Qi moderate-dose treatment reduced neutrophil infiltration by 44% compared to the arthritic group (Table 3). Bi-Qi treatment at high dose or MTX treatment did not affect neutrophil infiltration. Bi-Qi treatment at high or moderate dose reduced lymphocyte infiltration by 38% and 65%, respectively, compared to the arthritic group (Table 3). Bi-Qi treatment at high or moderate dose and MTX treatment reduced plasma cell infiltration by 45%, 62%, and 59%, respectively, compared to the arthritic group. Proliferation of synovial cell and fibrous tissue was remarkably higher in the arthritic group compared to the healthy group. Bi-Qi treatment at moderate dose and MTX treatment reduced synovial cell proliferation by 33% and 44%, respectively, compared to the arthritic group (Table 3).

**Table 3:** Pathological feature of synovial membranes

Group (*n* = 8)	Neutrophil infiltration	Lymphocyte infiltration	Plasma cell infiltration	Synovial cell proliferation	Proliferation of fibrous tissue
Healthy	0	0	0	0	0
Arthritic	1.00 ± 0.46	1.63 ± 0.35	1.81 ± 0.26	1.69 ± 0.37	1.38 ± 0.44
BQ high dose	0.56 ± 0.32	1.00 ± 0.53^*^	1.00 ± 0.53^**^	1.19 ± 0.26	0.94 ± 0.42
BQ moderate dose	0.44 ± 0.32^*^	0.56 ± 0.32^***^	0.69 ± 0.26^****^	1.13 ± 0.52^*^	0.75 ± 0.27^*, &^
MTX	0.50 ± 0.38	1.06 ± 0.42	0.75 ± 0.38^****^	0.94 ± 0.32^**^	1.31 ± 0.37

Data are presented as mean ± SD

*BQ* Bi-Qi capsule, *MTX* methotrexate

Significant effect of treatment compared to arthritic group, *P < 0.05, ^**^P < 0.01, ^***^P < 0.001, ^****^P < 0.0001; compared to MTX-group, ^**&**^*P < 0.05*

### Bi-Qi treatment alleviated arthritis-induced cartilage destruction and inflammatory cell infiltration in cartilage

**Fig. 5:**
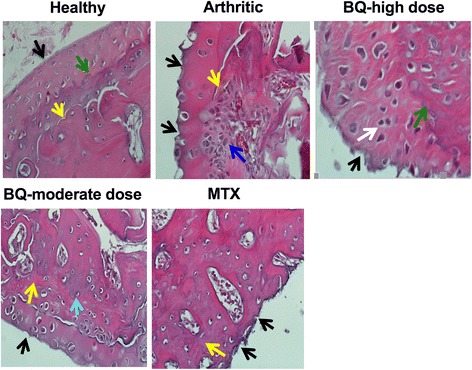
Bi-Qi capsule (BQ) or methotrexate (MTX) treatment alleviated collagen-induced arthritis (CIA)-induced cartilage destruction. Representative histological images of cartilage from the paw joint stained with H&E. Black arrow, cartilage surface; green arrow, subchondral bone; yellow arrow, bone trabeculae; blue arrow, chondrocytes; white arrow, osteoclast-like cell; light green arrow, osteoblast-like cell

Cartilage histology assessment showed a smooth and intact cartilage surface in the healthy group (Fig. 5), while CIA caused deterioration of the cartilage compared to the healthy group, as expected (Fig. 5). Bi-Qi moderate-dose treatment alleviated arthritis-induced cartilage destruction more efficiently compared to Bi-Qi high-dose treatment (Fig. 5). Cartilage damage depth and range, proliferation of fibrous tissue, and inflammatory cell infiltration in cartilage were significantly increased in the arthritic group compared to the healthy group (Table 4). Bi-Qi treatment at high or moderate dose and MTX reduced cartilage destruction depth by 52%, 63% and 52%, respectively, compared to the arthritic group. Bi-Qi treatment at high or moderate dose and MTX treatment reduced the extent of cartilage destruction by 55%, 70%, and 64%, respectively, compared to the arthritic group (Table 4). Similarly, Bi-Qi treatment at high or moderate dose and MTX decreased the proliferation of fibrous tissue in cartilage by 50%, 54%, and 50%, respectively, compared to the arthritic group. Moreover, Bi-Qi treatment at high or moderate dose and MTX treatment reduced inflammatory cell infiltration in cartilage by 22%, 54%, and 36%, respectively, compared to the arthritic group (Table 4). The inflammatory cell infiltration rate in the Bi-Qi moderate-dose treatment group was 41% lower compared to Bi-Qi treatment at high dose.

**Table 4:** Pathological features of cartilage

Group (*n* = 8)	Cartilage layer damage depth	Cartilage surface damage range	Proliferation of fibrous tissue	Inflammatory cell infiltration
Healthy	0	0	0	0
Arthritic	2.38 ± 0.52	2.50 ± 0.53	2.50 ± 0.53	2.75 ± 0.46
BQ high dose	1.13 ± 0.64^****^	1.13 ± 0.35^****^	1.25 ± 0.46^***^	2.13 ± 0.35^*^
BQ moderate dose	0.88 ± 0.35^****^	0.75 ± 0.46^****^	1.13 ± 0.35^****^	1.25 ± 0.46^****, &^
MTX	1.13 ± 0.35^****^	0.88 ± 0.64^****^	1.25 ± 0.46^***^	1.75 ± 0.46^***^

Data are presented as mean ± SD

*BQ* Bi-Qi capsule, *MTX* methotrexate

Significant effect of treatment compared to arthritic group, ^*^P < 0.05, ^**^P < 0.01, ^***^P < 0.001, ^****^P < 0.0001; compared to BQ high-dose group, ^**&**^*P < 0.05*

## Discussion

Therapies for RA are mostly aimed at reducing the pain, stiffness, and further progression of joint destruction and inflammation. TCM formula-based therapies for RA are popular in China. Combination therapy of TCM formula with DMARDs such as MTX has been reported to be more effective than DMARDs or TCM formula alone [5]. Bi-Qi is one of the commonly prescribed TCM formulas for RA treatment. However, Bi-Qi-mediated modulation of RA pathogenesis has rarely been investigated. In this study, we extensively studied the effect of Bi-Qi on the pathogenesis of CIA, and MTX treatment was used as a positive control. Bi-Qi alleviated local joint and systemic inflammation, immune cell infiltration to the synovium, synovial hyperplasia and cartilage destruction caused by CIA. The majority of features of CIA pathogenesis were alleviated by treatment with a moderate dose of Bi-Qi. The Bi-Qi treatment at moderate dose alleviated paw swelling, and reduced OPN in serum and synovium, the arthritic score, serum TNF-α, and fibrous tissue proliferation in the synovium more effectively compared to high-dose Bi-Qi. This is the first study to report that Bi-Qi alleviates the majority of RA symptoms in the CIA rat model.

Pro-inflammatory cytokines TNF-α, IL-6, IL-1β, and IL-18 are overexpressed in the joints in RA and play a vital role in the pathogenesis of RA [32, 33]. TNF-α activates the cytokine cascade in the joints in RA via stimulation of pro-inflammatory cytokines and inhibition of anti-inflammatory cytokines [34, 35]. TNF-α upregulation in RA occurs not only in joints and synovial fluid, but also in serum. Although TNF-α inhibitors are available to treat RA, around 30–40% of patients do not respond to this treatment [36–38]. Therefore, partial responders and non-responders to anti-TNF-α constitute a residual group with an unmet clinical need. In this study we found that moderate-dose Bi-Qi but not MTX treatment inhibited a rise in the TNF-α serum level in CIA. Our finding is in accordance with the findings from the clinical study by Nishina and colleagues who reported that MTX treatment is unable to reduce serum TNF-α in patients with early RA [39]. Similarly, we found that both moderate-dose Bi-Qi and MTX treatment slightly alleviated the rise in serum IL-18, another key player in RA pathogenesis. Rooney and colleagues reported that DMARD treatment in patients with RA slightly downregulates serum IL-18, but highly alleviates synovial IL-18 [40]. This indicates that the Bi-Qi might have potential to downregulate the pro-inflammatory cytokines in RA-affected joints.

COMP is a serum marker for disease activity in RA and a potential therapeutic target to treat RA [41]. Clinical studies have shown that DMARDs do not reduce serum COMP in RA [42]. Anti-TNF-α therapy slightly alleviates serum COMP (by 7.5%) in RA after 6 months of treatment [43]. Interestingly, we found that Bi-Qi remarkably reduced serum COMP (by 38%) in CIA. OPN is another protein highly expressed in RA, which regulates cytokine production in macrophages, dendritic cells, and T cells, and thereby activates the inflammation cascade [16, 17]. Clinical studies have shown that DMARDs and anti-TNF-α therapy in RA reduce serum OPN [44]. We found that Bi-Qi treatment at moderate dose remarkably reduced serum OPN (by 57%) in CIA. Moreover, MTX treatment efficiently reduced serum OPN. Bi-Qi treatment greatly reduced COMP and OPN in synovial and joint cartilage. Our results showed that Bi-Qi has the potential to reduce COMP and OPN expression in CIA.

DMARDs and other biological therapies reduce synovial hyperplasia, cartilage destruction, inflammatory cell infiltration, and disease activity score in RA [45]. We found that Bi-Qi treatment at moderate dose reduced the arthritic score, inflammatory cell infiltration, synovial hyperplasia, cartilage destruction, and pannus formation as effectively as MTX treatment. Bi-Qi is very cheap in comparison to MTX. Folic acid is usually prescribed to reduce the adverse effect of MTX. In the field of TCM, the combination of different TCM plant extracts is supposed to synergistically nullify the adverse effect of each other. Bi-Qi has been formulated based on the 3000 years of practice of TCM to minimize the systemic adverse effects. However, Bi-Qi treatment has an adverse effect on the gastrointestinal tract causing diarrhoea, which was occasionally observed in the Bi-Qi-treated rats. Besides this, no other systemic adverse effects of Bi-Qi treatment were observed. However, future studies focused on the possible adverse effect of Bi-Qi in vital organs and systems are highly essential. Among major compounds of Bi-Qi, salvianolic acid B is an antioxidant, glycyrrhizin has anti-autoimmune-reactive properties, brucine is an anti-inflammtory and analgesic compound, Strychnine is a convulsant, and tanshinone IIA is active against oxidative stress [7]. However, many other compounds present in Bi-Qi are still unknown. Identification of all compounds present in Bi-Qi, their metabolism and systemic adverse effects will provide the important insights to corroborate the use of Bi-Qi as a potential therapy for RA. In this study, moderate-dose Bi-Qi more efficiently alleviated CIA-induced inflammation, synovial hyperplasia, and cartilage destruction compared to high-dose Bi-Qi. For a clear explanation of this effect, further studies are needed focusing on the dose-dependent effect of Bi-Qi on molecular and cellular signaling of immune cells involved in RA pathogenesis.

## Conclusions

Bi-Qi alleviated local joint and systemic inflammation, synovial hyperplasia and cartilage distruction caused by CIA. Moderate dose Bi-Qi treated the majority of CIA pathogenesis. This provides fundamental evidence for the anti-arthritic properties of Bi-Qi and corroborates the use of Bi-Qi TCM formula for the treatment of RA.

## Additional file

Additional file 1: **Table S1.** Physiological and behavioral observation. **Table S2.** Body weight. (DOCX 18 kb)

## Abbreviations

Bi-Qi: Bi-Qi capsule
CIA: Collagen induced arthritis
COMP: Cartilage oligomeric matrix protein
DMARDs: Disease-modifying anti-rheumatic drugs
ELISA: Enzyme-linked immunosorbent assay
HPF: High-powered field
IL-18: Interleukin-18
MTX: Methotrexate
OPN: Osteopontin
PBS: Phosphate-buffered saline
RA: Rheumatoid arthritis
S.D rats: Sprague-Dawley rats
TCM: Traditional Chinese medicine
TNF-α: Tumor necrosis factor-alpha
