# Supplementary material for: Traditional Chinese medicine formula Bi-Qi capsule alleviates rheumatoid arthritis-induced inflammation, synovial hyperplasia, and cartilage destruction in rats
Source: Arthritis Res Ther. 2018 Mar 14;20:43. doi: 10.1186/s13075-018-1547-6 (PMC5853033; doi:10.1186/s13075-018-1547-6)
Supplement: Supplementary file 1 — Table S1. Physiological and behavioral observation. Table S2. Body weight. (DOCX 18 kb) [file 13075_2018_1547_MOESM1_ESM.docx]

**Table S1:** Physiological and behavioral observation

| Group | Walking  gait | Mental alertness | Hair  Color | Diet | Stool |
| --- | --- | --- | --- | --- | --- |
| Healthy | Free | Responsive | Neat | Normal | Normal |
| Arthritic | Limited | Insensitive | Messy | Decrease | Normal |
| BQ-high dose | Slow | Less responsive | Gloomy | Slightly decrease | Slightly loose |
| BQ-moderate dose | Slow | Less responsive | Gloomy | Slightly decrease | Slightly  loose |
| MTX | Slow | Less responsive | Gloomy | Slightly decrease | Slightly loose |

**Table S2:** Body weight

| Days | Healthy | Arthritic | BQ-high dose | BQ-moderate dose | MTX |
| --- | --- | --- | --- | --- | --- |
| 0 | 148.9±4.24 | 148.3±4.55 | 150.1±4.41 | 147.1±5.55 | 149.4±5.52 |
| 7 | 185.9±4.72**** | 154.6±4.72 | 160.3±4.85 | 155.5±4.7 | 154.8±3.68 |
| 14 | 203.4±10.16**** | 161.2±5.92 | 169.9±6.33 | 163.2±7.00 | 163.1±6.54 |
| 21 | 213.5±16.59**** | 170.3±13.25 | 180.4±9.57 | 170.8±6.80 | 168.9±6.08 |
| 28 | 222.4±25.76**** | 175.6±12.08 | 187.2±8.79 | 179.4±8.02 | 178.2±8.08 |
| 35 | 252.4±17.82**** | 182.2±12.44 | 193.9±10.41 | 190.2±10.65 | 187.3±9.06 |
| 42 | 282.3±32.09**** | 186.8±7.51 | 201.3±6.23 | 200.6±5.97 | 199.2±5.65 |

Data are presented as mean ± SD, n=8. Significant effect of control group compared to Arthritic, BQ-high dose, BQ-moderate dose and MTX group, *^****^* *P < 0.0001*. BQ, Bi-Qi capsule; MTX, Methotrexate.
